# Preparation of environmentally friendly acrylic pressure-sensitive adhesives by bulk photopolymerization and their performance

**DOI:** 10.1039/c9ra10514j

**Published:** 2020-03-10

**Authors:** Menglu Zhu, Zhanshuo Cao, Haijun Zhou, Yijun Xie, Guohua Li, Nongyue Wang, Yingchun Liu, Lianqi He, Xiongwei Qu

**Affiliations:** Hebei Key Laboratory of Functional Polymers, School of Chemical Engineering, Hebei University of Technology Tianjin 300130 P. R. China xwqu@hebut.edu.cn nkligh@126.com; Institute of Energy Resources, Hebei Academy of Sciences Shijiazhuang 050081 P. R. China; Jinghua Plastics Industry Co. Ltd. Langfang 065800 P. R. China; Engineering Centre of Flexible Special Hose of Hebei Province Hengshui 053500 P. R. China

## Abstract

Polyacrylic pressure-sensitive adhesives (PSAs) based on butyl acrylate (BA), 2-hydroxyethyl acrylate (HEA), and acrylic acid (AA) were prepared by a bulk polymerization process triggered by a radical photoinitiator under UV irradiation and UV-crosslinking. 1,6-Hexanediol diacrylate (HDDA) with difunctional groups was introduced into the PSAs to modify semi-interpenetrating network structures. The effect of HDDA content on the pressure-sensitive performance was comprehensively tested. The viscosity of the prepolymer was measured by a rotational viscometer. Prepolymers obtained by a photoinduced process and UV crosslinking process were confirmed *via* Fourier transform infrared spectroscopy (FTIR). All double bonds participated in the copolymerization without any remaining monomers, which reflected the concept of green environmental protection. Gel content in the crosslinked portion was examined by Soxhlet extraction, whilst the soluble molecular weight of PSAs was characterized by gel permeation chromatography (GPC). The viscoelastic properties of polymer films were determined by dynamic mechanical analysis (DMA). The *T*_g_ value and storage modulus (*G*′) of the PSAs were enhanced with the addition of HDDA. Moreover, three fundamental adhesive properties, *i.e.* loop tack force, peel force and shear strength of PSAs, were measured. The results showed that UV crosslinking technology achieved a good balance of the three forces with excellent pressure-sensitive properties.

## Abstract 2

Polyacrylic pressure-sensitive adhesives based on butyl acrylate, 2-hydroxyethyl acrylate, and acrylic acid were prepared by a bulk polymerization process triggered by a radical photoinitiator under UV irradiation and UV-crosslinking.

## Graphical abstract

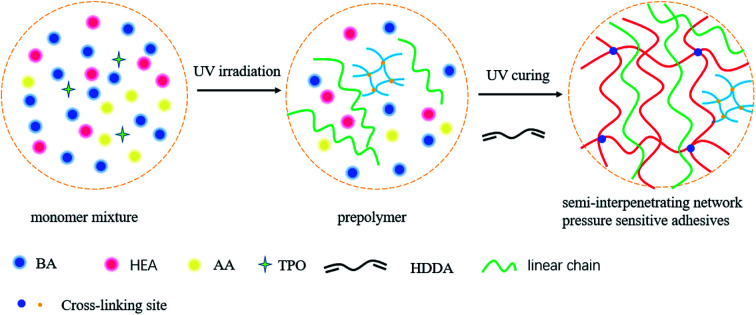

## 1 Introduction

Pressure-sensitive adhesives (PSAs) are viscoelastic materials that can be bonded to a wide range of solid surfaces, such as metallic and non-metallic surfaces, using slight pressure with a short contact time.^1^ The three basic properties of PSAs are tack, which is the ability to wet and adhere quickly on various substrates, peel strength which is the ability to resist removal by peeling, and shear resistance which is the ability to resist creep when shear force is applied.^2^ Of course, the performance of PSAs cannot be separated from their composition. The composition of polyacrylic PSAs is relatively simple, which involves three major acrylate monomers such as soft monomers with a lower glass transition temperature to provide tackiness, hard monomers with a higher glass transition temperature to provide cohesion, and functional monomers for modification *via* crosslinkers.^3,4^

Polyacrylic PSAs have been successfully applied in many fields including high-tech fields (*e.g.* automotive industry, electronic industry, and aerospace industry) and fields of our daily life (*e.g.* printing, tapes, labels, and a range of medical products) due to the inherent advantages.^5^ They have excellent transparency, optical performance, the ability to resist oxidation caused by sunlight. The proportion of polyacrylic PSAs is approximately 40% in the world market. Among them, solvent-based polyacrylic PSAs account for 50%, water-based polyacrylic PSAs account for 40%, and less than 10% of the solvent-free polyacrylic PSAs. Polyacrylic PSAs prepared by solution polymerization or emulsion polymerization have a reaction time of several hours, thus resulting in high energy consumption, and contain volatile organic compounds (VOCs) leading to increase costs for post-treatment and environmental pollution problems.^6^ On the contrary, UV technology can address these issues. It is divided into two parts: UV initiation and UV crosslinking.^7^ UV technology for preparing polyacrylic PSAs is emerging due to its environmental friendliness.^8^ Recently, a lot of attentions have been paid to the preparation of polyacrylic PSAs *via* photopolymerization due to its unique superiorities such as low cost, low energy consumption, no toxic catalysts, high efficiency and limited space.^9,10^ The reaction of forming polymers is very fast *via* a radical polymerization mechanism, which is no more than a few minutes.^11^ Therefore, it greatly shortens the crosslinking time. Moreover, reaction process is polymerized in bulk without solvent, which reduces the emission of organic volatiles. Currently, UV light has been applied in various fields such as coatings, adhesives and inks. Due to the limited penetration of light, it is not suitable for the formation of too thick products, which is the main reason for the limitation of ultraviolet light.^12,13^

However, linear molecular chains of polyacrylic PSAs may not be suitable for higher temperatures. Moreover, uncrosslinked structures of polymers can cause relative slippage between molecular chains. Therefore, in order to increase intermolecular force, it is necessary to inhibit the movement of the polymer chains. Pressure-sensitive properties and thermal stability of PSAs can be enhanced to form network structures by adding crosslinking agents. As we all know, crosslinking can be divided into three categories: (1) physical crosslinking between molecular chains formed by hydrogen bonds, which are reversible and poor; (2) chemical crosslinking through the reaction of functional groups in main chains; (3) semi-interpenetrating polymer networks named semi-IPNs.^14,15^ Functional groups present in the main chains such as carboxyl groups which requires external crosslinking agents to generate network structures. Common crosslinkers contain metal acid esters, metal chelates, polyfunctional isocyanates, metal salts, amino resins and UV crosslinkers. Semi-interpenetrating polymer networks consist of linear main chains and network structures derived from multifunctional monomers. The mechanism for the formation semi-IPNs is that the network structures of polymers pack linear backbones tightly.^16^ However, research on preparing acrylic prepolymer and polyacrylic PSAs both using UV irradiation technologies is scarce and not widely reported. Most investigations have only stayed at the UV crosslinking stage, but less research on the preparation of prepolymers at the UV initiation stage.^17–21^ The process without solvent is also the technology which has not been realized in our country as the limitations of equipment manufacture and the process engineering controllability. Czech *et al.* synthesized in ethyl acetate at boiling point of solvent during radical polymerization of acrylics mixture of photoreactive UV-crosslinkable solvent-borne acrylic PSA and investigated the influence of a relatively new class of unsaturated copolymerizable photoinitiators, thermal starter AIBN and copolymerizable photoinitiator 4-acryloyloxy benzophenone (ABP).^22^ However, the resultant UV-crosslinkable PSA needed to remove the solvent, and resulting the problem to recycle the solvent. To our knowledge, there is no reports about the preparation of PSAs using UV-initiation and crosslinking methods. In order to reduce energy consumption and become more environmentally friendly, this research is focused on bulk polymerization method for the preparation of PSAs by UV initiation. In this study, the polyacrylic PSAs based on butyl acrylate (BA), 2-hydroxyethyl acrylate (HEA), and acrylic acid (AA) were synthesized *via* bulk polymerization process triggered by a radical photoinitiator under UV irradiation and UV-crosslinking, as above monomers for polymerization were commercial and cheap. Emphasis was placed on the effects of different contents of cross-linking agents on the molecular structures and pressure-sensitive properties, and to achieve a good balance between adhesion and cohesion.

## 2 Experimental

### 2.1 Materials

Diphenyl(2,4,6-trimethylbenzoyl)phosphine oxide (TPO, Tianjin Jiuri Chemistry Co, China) was used as the photoinitiator during the polymerization stage without further purification. 1,6-Hexanediol diacrylate (HDDA, Shanghai Macklin, China) was purchased as the difunctional crosslinker to form network structures. *n*-Butyl acrylate (BA) obtained from Pulaihua Chemistry Agents of China was further purified after purchase. Firstly, they were washed three times with 2% NaOH solution to remove the inhibitor, followed by repeated washing with water for several times until neutral. Finally, monomers were dried with CaCl_2_ overnight. Acrylic acid (AA) and 2-hydroxyethyl acrylate (HEA) available from Shanghai Macklin were passed through a column of activated aluminum oxide to remove the inhibitors. 2-Hydroxy-2-methyl-1-phenylpropanone (1173) supplied by Tianjin Jiuri Chemical Co was used as the initiator of UV-crosslinking process.

### 2.2 Synthesis of acrylic PSA prepolymer

**Fig. 1:**
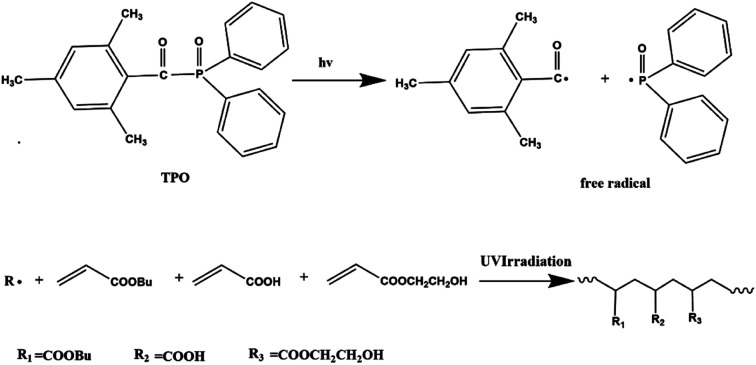
Schematic graph of photopolymerization for acrylic PSA prepolymer.

The environmentally friendly acrylic prepolymer was synthesized in bulk polymerization under UV radiation with TPO (0.1 g) photoinitiator. The scheme graph is shown in Fig. 1. BA (82.8 g), HEA (10 g) and AA (7.2 g) were prepared to form prepolymers. The monomers mixture and photoinitiator were placed in a 250 mL four-necked flask and stirred for 5 minutes. In order to eliminate the inhibition of oxygen, nitrogen was introduced during the photopolymerization process. The mixture containing monomers and initiator was exposed to UV LED lamp (TY-UV-8, Dongguan Tuoyi Electronic Co. Ltd.) with wavelength of 395 nm and the power of 9 W. As the viscosity of the polymer increased to a certain extent, the UV LED lamp was turned off. The polymer chains and unsaturated monomers were existed in the oligomers obtained.

### 2.3 Formation of polyacrylic PSAs

The mixtures used for UV-curable polyacrylic PSAs were prepared by blending acrylic prepolymer synthesized *via* UV irradiation and HDDA crosslinking agent with TPO (1 wt%) and 1173 (1 wt%) photoinitiators. The amount of HDDA crosslinking agent was 0, 0.1, 0.3, 0.5, 0.7, 0.9 phr (per hundred part of the prepolymer). The mixtures were stirred for 10 minutes and then removed air bubbles. In coating stage, mixtures with different HDDA contents were coated onto PET films (50 μm) with 100 μm thickness controlled by a coating device. The UV crosslinking process was carried out using an UV lamp from Philips Company (XP-5) with main wavelength of 365 nm and the power of 200 W. The UV exposure was characterized by a UV radiometer (UE500), provided by Xinhengsen Corporation. All samples were subjected to UV lamp for 20 s.

### 2.4 Characterization

#### 2.4.1 Prepolymer viscosity

The viscosities of prepolymers *via* UV LED radiation were tested using a Rotary Viscometer (NDJ-8S, Shanghai Fangrui Co.) at 25 °C.

#### 2.4.2 Fourier transform infrared spectroscopy (FTIR)

The spectra of prepolymers synthesized by UV LED radiation and after UV crosslinking process were obtained *via* Fourier transform infrared spectroscopy (Vector-22, Bruck Company, Germany). The spectral range was 400 to 4000 cm^−1^ with a resolution of 4 cm^−1^. All spectra were modified with carbon removal and baseline correction.

#### 2.4.3 Gel content and molecular weight measurements

The gel content of the acrylic PSA polymers was obtained *via* a Soxhlet extraction with boiling THF at 70 °C for 24 h. After refluxing, insoluble polymers were placed in a vacuum oven at 85 °C for 48 h until to a constant weight. The gel content of the PSAs was calculated according to the equation:1
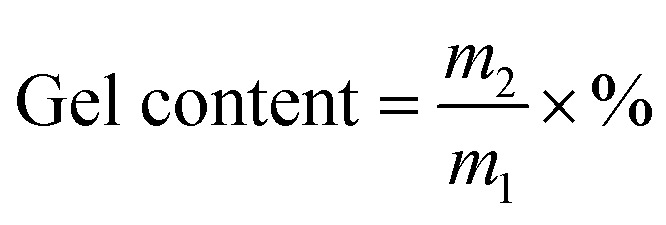
where *m*_1_ is the initial weight of the sample, *m*_2_ is the residual weight after removing the soluble polymer and drying in a vacuum oven at 85 °C for ten hours.

The molecular weight between cross-linking points (*M*_c_) was determined by the dry polymer swollen by toluene for two hours at room temperature. The detail information about *M*_c_ saw the ref. 23 and 24.

The molecular weight of the soluble fraction dissolved by THF such as *M*_n_, *M*_w_ and the molecular weight distribution (*M*_w_/*M*_n_) were measured by gel permeation chromatography (PL-GPC220, Polymer Laboratories Corporation, UK). THF was set as the flow phase with flow rate for 1 mL min^−1^.

#### 2.4.4 Dynamic mechanical analysis (DMA)

The viscoelastic properties of polyacrylic PSAs were characterized with a Triton 2000 (Key worth, UK) dynamic mechanical analyzer in plate clamp mode obtained the storage modulus (*G*′), tangent delta (tan *δ*) and glass transition temperature (*T*_g_). The thickness of the sample is *ca.* 1 mm. Samples were measured with a frequency of 1 Hz. The heating temperature ranged from −70 to 80 °C with the heating rate of 5 °C min^−1^. The glass transition temperature is the temperature where tan *δ* reaches the peak value.

#### 2.4.5 Pressure-sensitive adhesive performance

All adhesive tests were carried out at 23 ± 2 °C and 50 ± 5% relative humidity. Before testing, the samples were conditioned for 24 h at the standard condition. Loop tack and 180° peel testing were carried out with a universal tensile tester (CMT-6104, Shenzhen SANS Testing Machine Co., Shenzhen, China) on a stainless steel substrate. Test methods were in accordance with the FINAT test method numbers 9 and 1 at 300 mm min^−1^. The samples for peel force measurement were adhered to a stainless steel substrate and were rolled over three times with a 2 kg rubber roller. Shear resistance was done on a stainless steel substrate with a 25 × 25 mm^2^ PET coated strip and a 1000 g hanging weight according to FINAT test method number 8. The samples were rolled over three times with a 2 kg rubber roller. The holding time was recorded and used to evaluate shear resistance of polyacrylic PSAs. The average values were from five trials.

## 3 Results and discussion

### 3.1 Prepolymer viscosity

**Fig. 2:**
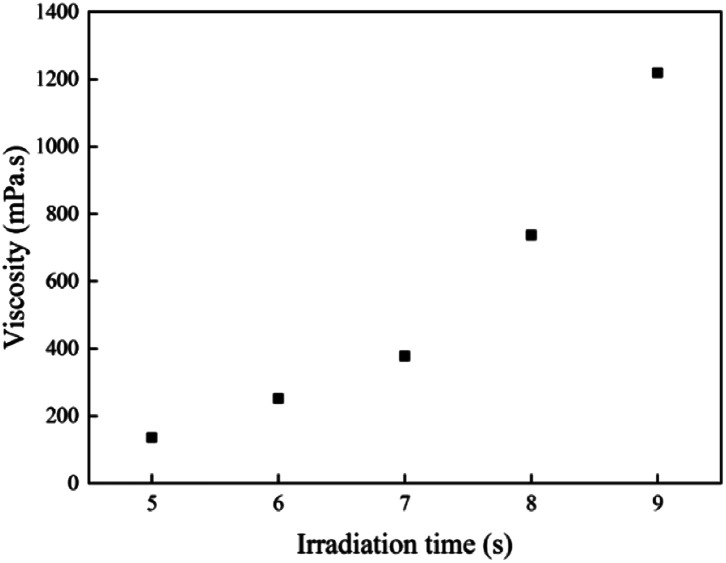
The viscosities of the acrylic prepolymers with different UV irradiation times.

The viscosity of the prepolymer is very important for coating on a substrate. The results of viscosity test at 25 °C for all samples with different irradiation times are shown in Fig. 2. Prepolymers with low viscosity prepolymer easily flow, while the ones with high viscosity can spread on the substrate difficultly. It might be very difficult to coat onto a substrate if the viscosity of the viscous mixture is >50 000 mPa or <100 mPa.^25^ The viscosity of the acrylic prepolymer syrup must be precisely controlled by the illumination time under UV LED photopolymerization. The viscosity of prepolymer was *ca.* 1200 mPa s for 9 s. Therefore, the illumination time was critical to the viscosity of the prepolymer. Fig. 2 shows that the viscosity of the acrylic prepolymer increases with increasing illumination time. At the beginning of UV irradiation, the viscosity of the prepolymer was lower due to the slower decomposition of the photoinitiator. As the UV irradiation time increased, the number of free radicals increased. The monomers participated in copolymerization, which was equivalent to an increase in the solid contents, and thus the viscosity of polymers increased.

### 3.2 Fourier transform infrared spectroscopy (FTIR)

**Fig. 3:**
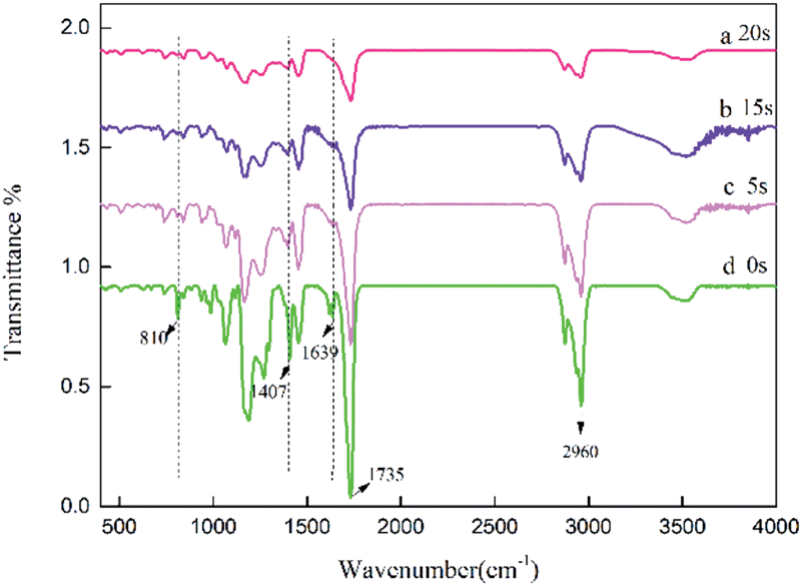
FTIR spectra of the acrylic prepolymers with different UV crosslinking times, (a) 0, (b) 5, (c) 15 s, (d) 20 s.

The specific peaks of polyacrylic PSAs with different UV crosslinking times were monitored by Fourier transform infrared spectroscopy (FTIR). Therefore, it was used to assess the reaction of monomers. The spectra of the prepolymer at different crosslinking times are shown in Fig. 3. The spectrum of the prepolymer is obtained by photopolymerization shown in curve (a) without the addition of crosslinker. The reaction of HDDA with the remaining monomers *via* UV crosslinking was also investigated. The spectra obtained at different crosslinking times with 0.9 wt% HDDA crosslinker are exhibited in curves (b)–(d). Most peaks of samples at different crosslinking times were similar. The sharp peaks observed at 1639 cm^−1^, 1407 cm^−1^ and 810 cm^−1^ can be attributed to the strong absorbance of C=C bonds. With the increasing crosslinking time, the intensities of these peaks decreased from curve (a), to curve (c), which was ascribed to the reaction of C=C bonds. When the crosslinking time reached 20 s, the C=C absorption peaks disappeared, indicating that all monomers with double bonds reacted completely in curve (d). The change in the absorption intensity of the C=C bond illustrated that UV crosslinking process occurred.

### 3.3 Gel content and molecular weight of polyacrylic PSAs

**Fig. 4:**
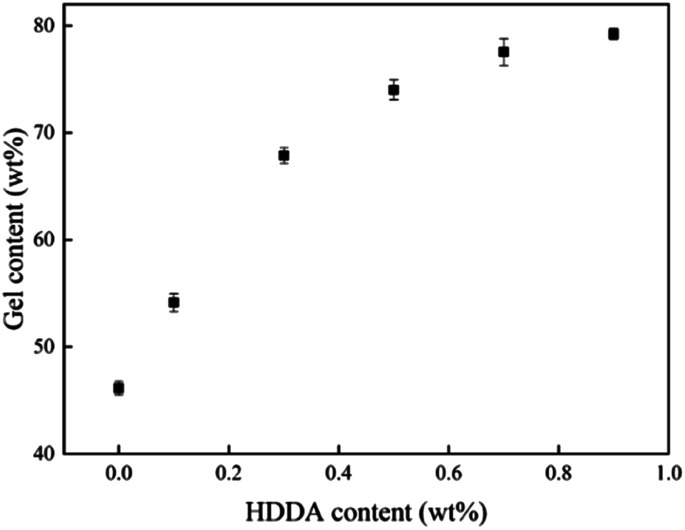
Gel content of the polyacrylic PSAs with HDDA content.

The relationship between the gel content of acrylic PSAs polymer and HDDA content is shown in Fig. 4. The average molecular weights between crosslinking points (*M*_c_) at different HDDA contents are listed in Table 1. As can be seen from Fig. 4 and Table 1, the gel content increased with the increase of HDDA content, but the *M*_c_ had an opposite trend. This phenomenon is well known for the polymerization of BA and BA-dominated co-monomer systems.^26^ The gel arose from termination by the coupling of propagating long-chain branches from intermolecular or intramolecular chain transfer to polymer.^2^ In this case, a three-dimensional continuous network structure was created in acrylic PSAs polymer after the reactions between the C=C in the polymer branches. Apparently, the number of crosslinking sites increased with the content of HDDA, and correspondingly, the cross-linking network structures became denser. Therefore, the gel content increased and *M*_c_ decreased. After the addition of HDDA, the gel content increased rapidly, and reached 80% when the HDDA content was only 0.9 wt%. Therefore, the final PSA structure contained 45 wt% from the self-crosslinked content of the monomers added (when HDDA content was 0%), which resulted from the hydrogen abstraction and chain transfer reactions of a number of tertiary carbon atoms of reactive monomers, such as butyl acrylate (BA), 2-hydroxyethyl acrylate (HEA), and acrylic acid (AA).^24,27–30^ Other PSAs were composed of 55 wt% to 80 wt% crosslinked contents when the HDDA crosslinker content increased from 0.1 wt% to 0.9 wt%. The remainders were soluble acrylate copolymers.

**Table 1:** Summary of the molecular weights of the polyacrylic PSAs

HDDA (wt%)	0	0.1	0.3	0.5	0.7	0.9
*M*_c_ (g mol^−1^) × 10^−4^	2.60	1.91	1.27	0.92	0.66	0.19
*M*_n_ (g mol^−1^) × 10^−4^	3.36	3.17	2.82	2.74	2.42	2.32
*M*_w_ (g mol^−1^) × 10^−4^	9.76	8.55	8.01	7.72	7.16	6.84
MWD	2.91	2.70	2.80	2.82	2.96	2.64

The summary of various molecular weight parameters of the soluble PSA polymer at different HDDA contents is presented in Table 1 also. It can be seen that the molecular weights (*M*_n_, *M*_w_) of the soluble polymers decreased with the increase of HDDA content. The reason for this behavior was that the decrease of the soluble polymer molecular weight was due to a transfer from the soluble fraction to the gel of large branched polymer chains formed by chain transfer to the polymer and termination by combination reactions, while had no significant effect on the soluble MWD.

### 3.4 Viscoelastic properties

**Fig. 5:**
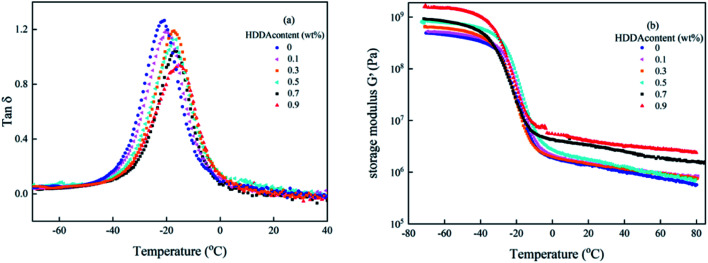
Variation of (a) tan *δ* and (b) *G*′ with the temperature of the polyacrylic PSAs with different HDDA contents.

Dynamic mechanical analysis (DMA) reflects the molecular motions of the viscoelastic materials under the periodic forces. Plots of tan *δ* and storage modulus *G*′ as a function of temperature for polyacrylic PSAs are obtained by DMA shown in Fig. 5. From Fig. 5(a), it can be seen that for all curves only one glass transition temperature peak existed and the peak width did not change significantly. These results demonstrated that random copolymers were formed among the reactant components. The glass transition temperature (*T*_g_) of the PSA polymer increased with the increase of the HDDA content due to higher crosslinking density presented in Table 2. This phenomenon was explained that the formation of a crosslinked network limited the movement of the linear backbone. The storage modulus indicated that the stiffness and the ability of resistance to deformation of the material increased. From Fig. 5(b), it can be seen that *G*′ values increase with increasing HDDA content.

**Table 2:** Viscoelastic parameters for polyacrylic PSAs with different HDDA contents

HDDA (wt%)	*M*_e_ (g mol^−1^) × 10^−4^	*T*_g_ (°C)	*G*′ (MPa) at 25 °C	tan *δ* at 25 °C
0	2.19	−21.08	1.22	0.018
0.1	2.02	−18.56	1.33	0.014
0.3	1.88	−17.78	1.61	0.013
0.5	1.64	−16.71	1.39	0.011
0.7	0.85	−14.75	3.16	0.009
0.9	0.69	−13.51	3.81	0.006

The entanglement molecular weight (*M*_e_), which is the average molecular weight of the molecular chain between two entangled nodes in the polymer, can be calculated as follows:^19,31^5
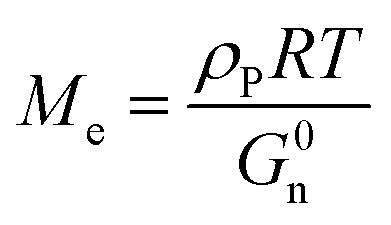
where *ρ*_P_ refers to the density of the polymer (about 1.06 g cm^−3^ for acrylic polymer), *R* is the gas constant being equal to 8.31 J mol^−1^ k^−1^, *T* is the absolute temperature where *G*^0^_n_ is located, and *G*^0^_n_ is determined from *G*′ at the onset of the rubbery region (usually where tan *δ* reaches a minimum after a prominent maximum).

*M*_e_ values are presented in Table 2 also. It was clearly founded that *M*_e_ decreased with the increase of HDDA content, which was attributed to the increasing degree of entanglement of the polymer chain. These DMA data listed in Table 2 that highlight the high sensitivity of the viscoelastic properties to the details of the crosslinked polymer network architectures are useful to further analyze the adhesive properties of the PSA polymer at room temperature as discussed below.

### 3.5 Pressure-sensitive adhesive properties

The adhesive properties of polyacrylic PSAs are commonly assessed by three indicators, *i.e.* loop tack, peel force and shear resistance. The values of these three indicators with HDDA content are listed in Table 3. From the results, it was obviously observed that the loop tack force and peel force decreased with the increase of HDDA content slightly, while the shear resistance increased dramatically.

**Table 3:** Effect of the HDDA content on the adhesive properties

HDDA content (wt%)	Tack force (N)	Peel force (N/25 mm)	Shear resistance (min)
0	19.39 ± 0.24	22.68 ± 0.24	1220 ± 160
0.1	19.02 ± 0.24	24.82 ± 0.25	2170 ± 230
0.3	18.75 ± 0.17	20.80 ± 0.27	2890 ± 210
0.5	16.20 ± 0.35	19.98 ± 0.15	4350 ± 250
0.7	15.02 ± 0.15	19.12 ± 0.22	6280 ± 280
0.9	14.78 ± 0.28	18.64 ± 0.22	7420 ± 220

#### 3.5.1 Loop tack force

The strength of an adhesive bond is determined by the thermodynamic contributions to the interfacial energy (van der Waals interactions, electrostatic forces, and hydrogen bonding) and the rheological contributions due to the viscoelastic dissipation during deformation of the polymer chains in the adhesive layer. In a tack test, the work of adhesion is dominated by this viscoelastic contribution.^32^Table 3 presents the loop tack forces of the PSAs with different HDDA contents. There may be two reasons to explain the decrease of the loop tack force. Firstly, the decrease of *M*_c_ of polyacrylic PSA with HDDA content indicated that an increasingly dense crosslinking network was created in the PSA, resulting in the decrease of the deformation ability of the polymer chains, which further weakened the bonding between the PSA and substrate. Secondly, considering the dynamic mechanical properties of the PASs, in order to deform rapidly within the timescale of the adhesive formation, a high viscosity/elastic modulus ratio (tan *δ*) at the frequency (1 Hz) and temperature (25 °C) of bonding formation for PSA polymer is necessary.^33^ Yang and Chang reported that the adhesive properties of the PSA polymer had a strong dependence on the loss factor tan *δ*.^34^ For the PSA polymer, the larger the tan *δ*, the higher the loop tack force, and the polymer is easier to deform and spread on the surface of the substrate. According to the DMA data displayed in Table 2, as the content of HDDA increased from 0 to 0.9 wt%, the value of tan *δ* at room temperature decreased from 0.018 to 0.006, resulting in lower adhesive properties. In addition, the increasing *T*_g_ and *G*′ also manifested that the rigidity of molecular chains increased with the increase of the HDDA amount. Therefore, the flexibility of the polymer chains was limited, and the loop tack force of the polyacrylic PSAs decreased as expected.

#### 3.5.2 Peel force

**Fig. 6:**
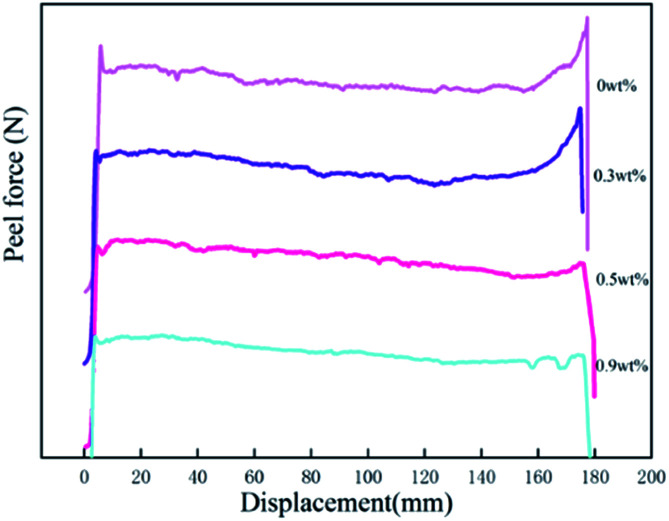
Force–displacement plot for the peel measurement.

Surface interactions play an important role in PSA bonds. The adsorption of the adhesive molecules onto the adherent surfaces occurs mainly by physical adsorption. Yang found that for polyacrylic samples, the surface tensions were in the range between 31 and 37 dyne cm^−1^.^35^ Because the surface tension of the stainless steel is at 44 dyne cm^−1^, we can expect good wetting to be achieved for all samples. The values of peel forces with different HDDA contents for the PSAs are also shown in Table 3. The peel strength of the polyacrylic PSAs decreased with the increase of HDDA content. The force measured during peel is composed of two components: the force required to overcome the work of adhesion, that is, to break the adhesive/substrate interfacial bond; and the force requires to deform the bulk of the adhesive.^35^ The force measured during the peel process is also the outcome of the viscoelastic process as tack force. To achieve a high fracture energy during separation of the adhesive bond, the PSA has to form bridging fibrils, and that these bridging fibrils can form only when the elastic modulus is below a certain value. As shown in Table 2, the soluble polymers having broad polydispersity (MWD: 2.64–2.96) are effective in providing high viscoelastic energy dissipation during peeling.^27^ A high molecular weight (*M*_n_, *M*_w_) was needed for the PSA film to undergo fibrillation during the peeling process. The increase in modulus arising from crosslinking acrylic polymers with HDDA component resulted in a lower peel strength, as shown in Table 2 and Fig. 5(b). As the modulus of the polyacrylic PSA increased, the amount of adhesive filamentation at the locus of failure decreased, and hence the volume of adhesion under deformation decreased. The amount of filamentation at the locus of failure would decrease as the modulus of the polyacrylic polymer increased. Fig. 6 shows graphically the relationships between the peel force and displacement for the adhesives as HDDA contents were 0, 0.3, 0.5, and 0.9 wt%, respectively. The failure modes for PSA films were adhesive failure. The increment in the HDDA content resulted in a decrease of the fibrillation during the peeling process.

#### 3.5.3 Shear resistance

UV photopolymerization of low-*T*_g_ polyacrylic PSAs carried out to complete conversion and produced a significant amount of microgels because of the chain transfer to the polymer *via* the hydrogen abstraction of tertiary vinyl carbons and crosslinking reaction.^36^ As the mode of failure in shear testing was cohesive for all PSAs, this suggested that the interior structures of PSAs played an important role in determining the shear resistance of the PSA film. From Table 3, it can be seen that the polyacrylic PSAs without HDDA has inferior cohesive strength, only about 1200 min of holding time, and the sample exhibited cohesive failure. However, the cohesive strength of the polyacrylic PSAs was greatly improved after cross-linking, with which the sample reached 2170 min with addition of 0.1 wt% HDDA content. It was showed in a previous research that gel content and network morphology had significant roles in influencing the shear resistance.^32^ In one aspect, as HDDA content increased, the gel content increased significantly (as shown in Fig. 4), leading to the larger shear resistance. The result was in good agreement with the work of Qie,^37^ who found that tack and peel strength decreased, while shear resistance increased with the increase in gel content. The most important factor was the increase in the polymer's *T*_g_ as a consequence of the increasing content of HDDA. A rise in *T*_g_ toward the testing temperature caused a reduction in polymer chain mobility and hence an increase in shear resistance. The other important reason responsible for the increase of the shear resistance came from the microstructure of the dried film, *i.e.* the entanglement molecular weight, *M*_e_. The chain ends of the micro-networks could entangle with the soluble polymer chains, which could entangle with chain ends of other particles after film formation. Chain entanglements behaved as pseudo-crosslinks that eventually disentangled under shear stress, but contributed to the measured shear resistance. It can be seen from the results in Table 2 that the decrease of the *M*_e_ resulted in the increase of the packing density of the molecular chains. Thus, a greater number of entanglements inhibited elongation of the macromolecular chains and improved the shear resistance of PSA. These results were very interesting and useful. Generally, the increase in shear resistance will dramatically decrease in loop tack force and peel force. For example, when the shear resistance increased from 1200 min to 4500 min, the loop tack force decreased from 9.53 N to 3.56 N and peel force decreased from 9.84 N/25 mm to 2.54 N/25 mm. At this very moment, the 1,4-butanediol dimethacrylate (BDDA) crosslinker content increased from 0 to 1 wt%.^24^ So was the results of the ref. 27 using poly(methyl methacrylate) as a physically crosslinker. For the present UV polyacrylic PSAs, when the HDDA content increased from 0 to 0.9 wt%, the shear resistance increased from 1220 min to 7420 min, while the loop tack force and peel force kept higher values, 14.78 N and 18.64 N/25 mm. Therefore, three properties for pressure-sensitive adhesives such as tack, peel strength and shear resistance were achieved with a balanced relationship.

## 4 Conclusion

By means of UV LED photopolymerization and UV crosslinking reaction with difunctional monomer (HDDA) for the modification of acrylic prepolymer and PSA, the balanced adhesive properties can be obtained by controlling the content of crosslinking agents for a wide range of applications. The process was eco-friendly, energy-saving and high-efficiency. Crosslinking agent contents affected the adhesive properties of polyacrylic PSAs by changing their molecular structures and viscoelastic behaviors. The loop tack and peel strength of the PSAs reduced slightly with the increase of the HDDA content, while the shear resistance of the PSAs increased dramatically. When the HDDA content was 0.9 wt% and the UV crosslinking time was only 20 s, the shear resistance of the PSA reached 7420 min, while the loop tack and 180° peel forces kept 14.78 N and 18.64 N/25 mm. The method in this study may be facilely used in practical applications.

## Conflicts of interest

There are no conflicts to declare.

## Supplementary Material
